# No Matter Your Age, “It’s Your Life, It’s Your Choice”: Compulsory Persuasion and Informal Rehabilitative Support in Youth and Adult Intensive Supervision Programs

**DOI:** 10.1177/0306624X241246514

**Published:** 2024-04-10

**Authors:** Sam Ghebrai, Dale Ballucci

**Affiliations:** 1 University of Western Ontario, London, Canada

**Keywords:** intensive supervision programs, rehabilitation, young offenders, adult offenders, coercion

## Abstract

Although existing literature identifies that public protection and risk reduction are the primary goals of intensive supervision programs (ISP), little is known about how or whether rehabilitation of high-risk offenders is prioritized outside of enforcing court-mandated conditions. Using qualitative semi-structured interviews and focus groups within two Canadian metropolitan ISPs, our study explores how rehabilitative support is offered to youth and adult high-risk offenders in the absence of formal conditions. Using the framework of late-modern rehabilitation and compulsory persuasion, we draw on themes of offender responsibilization and coercion to interrogate the provision of informal rehabilitative support. Our findings indicate that officers negotiate “voluntary agreements” with select high-risk offenders, which hierarchicalizes them into two groups: those worthy of informal support and those who “choose” not to want to rehabilitate. We also find that youth and adults are treated similarly despite substantive differences between the types of crimes committed.

## Introduction

Western criminal justice policies favor preventative and rehabilitative treatments for young offenders^1^ (R. Miller & Applegate, 2015; Myers, 2003). The underlying philosophy of this approach is one of “child saving,” where the best interests of youth are prioritized to minimize the harm experienced within the criminal justice system (Adorjan & Ricciardelli, 2018; Blevins et al., 2007; Cullen et al., 2007; Myers, 2003). However, in the context of serious or violent offenders, the distinctions fade between the criminal justice system’s treatment of youth and adults—especially for youth who commit “adult crime” (Bolin & Applegate, 2016; R. Miller & Applegate, 2015; Myers, 2003). Also known as high-risk offenders,^2^ the consequence for youth and adults who commit serious or violent crimes is placement into intensive supervision programs (ISPs)^3^ (Weinrath et al., 2016; Werth, 2017).

Over the past several decades, ISPs have increasingly been relied upon to identify and surveil high-risk offenders to prevent them from (re)offending (McCahill & Finn, 2013; Werth, 2017). Operating under the principles of deterrence and incapacitation, high-risk offenders who (re)commit a crime will be gradually met with harsher sanctions, including (re)incarceration (Canton, 2012; Hyatt & Barnes, 2017; Paparozzi & Gendreau, 2005; Schwalbe & Maschi, 2009). Although not always a priority of ISPs, in the era of late-modern rehabilitation (Robinson, 2008), a renewed emphasis is placed on balancing the punitive elements of the criminal justice system with rehabilitative elements (Adorjan & Ricciardelli, 2018; McNeill, 2014; Robinson, 2008). These contemporary shifts in criminal justice policies prompted ISPs to incorporate rehabilitative support—primarily by enforcing court-mandated recognizance conditions (Humphrey & van Brunschot, 2015; McCahill & Finn, 2013). A growing body of literature finds that discretionary decision-making by police, probation, and correctional officers not only impacts if and how court-mandated conditions are enforced but also how officers subvert such conditions to support offenders under their supervision (Ballucci, 2012; Haqanee et al., 2015; Kras et al., 2021). Despite such advancements, there is a dearth of research that examines how rehabilitation is facilitated in ISPs outside the enforcement of court-mandated conditions, and even less is known about youth ISPs (DuBois, 2022; Skeem et al., 2007; Weinrath et al., 2016). Addressing this gap, our research explores and compares how youth and adult ISPs provide informal rehabilitative support for high-risk offenders under their supervision.

This study draws on five qualitative semi-structured interviews and two focus groups with police officers^4^ and crime analysts from two ISPs in a Canadian metropolitan city. One ISP monitors young offenders between 12 and 24, and the other monitors adults aged 18 and older.^5^ Our research questions ask: (a) how ISPs initially approach the high-risk offenders under their supervision, (b) how they negotiate rehabilitative support in the absence of court-mandated conditions, and (c) what are the types of informal support that ISP officers provide? Across the youth and adult ISPs, participants express that voluntary agreements between themselves and the high-risk offenders are the foundation for providing informal rehabilitative support. Using the frameworks of late-modern rehabilitation (Robinson, 2008) and compulsory persuasion (Raynor, 1978; Vanstone, 2013), we draw on themes of offender responsibilization and coercion to interrogate the nature of the voluntary agreements used to provide informal rehabilitative support. Traditionally discussed in the context of probationary practice and social work, compulsory persuasion refers to the blurring of lines between consent and coercion when inducing compliance (Raynor, 1978; Vanstone, 2013). Contextualizing compulsory persuasion through the dominant late-modern narratives of rehabilitation, we examine the strategies officers and crime analysts deploy to induce compliance and whether strategies differ between youth and adult programs.

## Late-Modern Rehabilitation

Rehabilitation remains an essential function of the criminal justice system despite subordination to other punitive goals such as crime prevention and incapacitation (Adorjan & Ricciardelli, 2018; Garland, 2001; Lynch, 2000). Surviving and evolving in the 21st century, rehabilitation coupled itself in a “new discursive alliance with punitiveness” to maintain its legitimacy (Robinson, 2008, p. 435; see also Adorjan & Ricciardelli, 2018; Garland, 2001). Robinson (2008) characterizes this transformation as late-modern rehabilitation, and an increasing body of scholarship examines how punishment and rehabilitation are negotiated in this context (Adorjan & Ricciardelli, 2018; McNeill, 2014; Tidmarsh, 2020). In the late-modern era, rehabilitation and risk management are interconnected; actuarial tools are used to evaluate the levels of risk posed by an offender, thereby providing practitioners the ability to identify individuals who need more intensive rehabilitative support or those who require less intervention (Hannah-Moffat, 2005; Robinson, 2008).

Rehabilitation today is framed as a benefit for society rather than just for the offender, and through offender rehabilitation it can accomplish the goals of public protection and risk reduction (Hannah-Moffat, 2005; Humphrey & van Brunschot, 2015; McNeill, 2014; Robinson, 2008). For example, Canadian researchers find that post-sentencing supervision of sex offenders aids both in their rehabilitation and reintegration while simultaneously addressing the broader public’s concerns around safety and security (Humphrey & van Brunschot, 2015). However, late-modern scholarship expresses that this trend toward rehabilitation is an extension of punishment itself (Humphrey & van Brunschot, 2015; McNeill, 2014; Robinson, 2008; Tidmarsh, 2020). With the intertwined relationship between rehabilitation and punishment, the offender is responsible to desist from offending; otherwise, they continue to face increasing enforcement and rehabilitative interventions (Garland, 2001; Robinson, 2008). Situating rehabilitation in the late-modern context, thus, is crucial to understanding who is deemed worthy of rehabilitative support, what types of support they receive, and how compliance is induced.

## Compulsory Persuasion: Comparing Compliance Strategies Between Young and Adult Offenders

Offender compliance is a critical function in probation, parole, and intensive supervision programs and is inextricably linked to the effectiveness of a program (Bottoms, 2001; Canton, 2012; Robinson & McNeill, 2008; Schwalbe & Maschi, 2011; Schwartz et al., 2017; Skeem et al., 2009; Vanstone, 2013). Yet, the explicit ambiguity and tension between officers’ interpretations of their roles and responsibilities leads to drastically different compliance strategies (Ballucci, 2008; Haqanee et al., 2015; Kemshall & Maguire, 2001; Werth, 2018). In youth justice contexts, scholars find that, depending on their disposition, officers draw on a balance of enforcement tactics and therapeutic approaches to induce compliance (J. Miller, 2015; Schwalbe & Maschi, 2011; Schwartz et al., 2017). Research suggests that officer dispositions remain mixed for adult offenders, with enforcement-heavy approaches being more common (J. Miller, 2015; Skeem et al., 2007; Skeem & Manchak, 2008). Across both young and adult offenders, an abundance of scholarship suggests that balanced approaches are more effective in lowering recidivism rates (J. Miller, 2015; Paparozzi & Gendreau, 2005; Schwalbe & Maschi, 2011; Schwartz et al., 2017; Skeem & Manchak, 2008). Balanced approaches facilitate stronger relationships between an offender and the supervising officer while increasing offender compliance (J. Miller, 2015; Schwalbe & Maschi, 2009, 2011; Skeem et al., 2007). However, the very nature of this type of relationship is based on a blend of consent and coercion, otherwise known as compulsory persuasion (Raynor, 1978; Vanstone, 2013; Weaver & Barry, 2014). Compulsory persuasion has implications for the effectiveness of offender management programs, as this late-modern approach to rehabilitation shifts the responsibility of compliance from the officer into the hands of the offender (Vanstone, 2013).

Until the 1980s, enforcing compliance with recognizance conditions was primarily the probation officer’s duty, where it was their responsibility to maintain frequent contact with the offender (Vanstone, 2013). With the rapid advancement of surveillance technologies and the proliferation of various probationary programs, compliance responsibility has now shifted to the offenders (Vanstone, 2013; Weaver & Barry, 2014). Regardless of age, young and adult offenders must now make choices regarding compliance during their time on probation (Canton, 2012; Robinson & McNeill, 2008; Schwartz et al., 2017; Vanstone, 2013). However, their choices are effectively induced through the coercive tactics of supervising officers (Bottoms, 2001; Canton, 2012). Individuals are responsibilized for making decisions with the looming threat of incapacitation as motivation, blurring the line between consensual choice and coercion (Schwartz et al., 2017; Vanstone, 2013; Weaver & Barry, 2014). Considering the criminogenic factors that may lead to an individual’s criminality, responsibilization—or do-it-yourself rehabilitation—is inherently problematic (Canton, 2012; Gray, 2005; Phoenix & Kelly, 2013; Weaver & Barry, 2014). For young offenders, responsibilization dismisses the underlying economic, political, or social factors that contribute to their initial or repeated criminality (Goldson & Muncie, 2006; Phoenix & Kelly, 2013; Skeem et al., 2009). For adult parolees, Lynch (2000, p. 60) suggests that offender responsibilization imposes “unrealistic normalizing demands,” and when these demands are not met, they face “unforgiving consequences.” Instead, critics argue that it must be the officer’s responsibility—or at least a shared responsibility—to remove obstacles of potential offender non-compliance (Canton, 2012; Phoenix & Kelly, 2013; Weaver & Barry, 2014). Otherwise, the efficacy of programming falls under question.

## Informal Rehabilitation in Intensive Supervision Programs

Depending on the ISP, officers may provide rehabilitative support through both formal and informal means (Bouffard & Bergseth, 2008; Kras et al., 2021; Skeem et al., 2007; Weinrath et al., 2016). Formal rehabilitative measures include enforcement of court-mandated conditions such as mandatory drug and/or alcohol treatments or meetings with counsellors, psychologists, or therapists (Lecoq et al., 2021; Skeem et al., 2007). Informal measures, however, are not as clear-cut due to a dearth of data on what these interventions are or how they are implemented (Hyatt & Barnes, 2017; Weinrath et al., 2016). When courts do not impose specific rehabilitative conditions on a high-risk offender, an ISP officer’s discretion becomes the most robust way to provide rehabilitation. Research on informal support defines mentorship as one tactic that ISP officers employ to help rehabilitate young, high-risk offenders (DuBois, 2022; Weinrath et al., 2016). Compassionate officers’ mentoring reduces the likelihood of reoffending by supporting the pro-social behaviors of high-risk offenders (Bouffard & Bergseth, 2008; Weinrath et al., 2016). Conversely, if an officer only makes infrequent contact or is not interested in the high-risk offender’s success, the likelihood of reoffending increases (Chamberlain et al., 2018).

One important caveat, though, is that informal support can only be applied voluntarily since both the supervising officer and high-risk offender must tacitly agree to the terms of support (Bouffard & Bergseth, 2008; Canton, 2012; Vanstone, 2013). Reflecting compulsory persuasion, the onus is placed on the offender to comply with the voluntary agreement since, unlike formal measures, there are no enforceable mechanisms on behalf of the officers (Vanstone, 2013). As long as the offender is adequately supported, the effectiveness of voluntary agreements can be similar to mandatory treatments (Bouffard & Bergseth, 2008; Canton, 2012; McSweeney et al., 2007). The fundamental problem preventing such support is the detachment between supervising officers and the offenders (Robinson & McNeill, 2010; Vanstone, 2013; Ugwudike, 2010). Instead, rehabilitation should be a “collaborative contractual relationship” between the two parties (Vanstone, 2013, p. 21; see also Weaver & Barry, 2014). In the absence of such collaboration, high-risk offenders run the risk of remaining stuck in the perpetual cycle of criminality (Weaver & Barry, 2014). Our study, investigating how officers and crime analysts in youth and adult ISPs develop relationships with high-risk offenders, demonstrates that compliance is achieved through negotiating and providing informal rehabilitative support. Our analysis shows coercion and offender responsibilization play critical functions in both ISPs.

## Methods

This study draws on five semi-structured interviews and two focus groups with officers and crime analysts from two ISPs in one Canadian province. Data was collected over 3 months—the scope of the questions aimed to understand the process of managing high-risk offenders. Questions concerned participants’ positions within the agencies, length of employment, and details about their daily work. Follow-up interviews were scheduled during the 3 months. The interviews and focus groups varied between 30 minutes and 3 hours—with the focus groups running for 2 to 3 hours; each was recorded, professionally transcribed, and securely stored. For anonymity, neither the city, the province, nor the names of participants are identified; the participants are given pseudonyms. Aside from punctuation, the quotes used in the transcriptions have not been edited or altered.

## Data Collection and Analysis

The interviews and focus groups took place at the police station of each respective ISP. The two ISPs will hereafter be distinguished as the “youth program” and “adult program.” The participants were selected through consultations with the directors of each program. Altogether, ten variously ranked police officers and two crime analysts gave in-depth insights into their roles and responsibilities within the programs. The youth program consists of 12 employees, 4 of whom were interviewed for this study: the acting sergeant, the crime analyst, and two police constables. All the participants were highly experienced and provided detailed responses due to the length of their employment. The adult program was changing supervision at the time of the interviews. Including the incoming and outgoing sergeants, the adult program only consisted of eight employees. We conducted two focus groups and one interview with all eight of them. This included incoming and outgoing sergeants, the crime analyst, and the constables. Insights from the incoming sergeant were not included in this analysis because they were not officially part of the program. Comments from some focus group participants were also not included in the analysis because they either nodded in agreement to other officers’ remarks or did not contribute additional insights.

**Table 1.** Comparison Between the Youth and Adult ISPs Approaches.

Aspect	Youth ISP approach	Adult ISP approach	Comparison
Initial interaction	Informal, with an emphasis on monitoring conditions and offering help if possible	Informal, with a strong focus on deterrence and incapacitation	Both approaches start with enforcement strategies but offer rehabilitative support
Types of crimes warranting intensive supervision	Drug-related offences; physical assault; sexual assault; armed robberies; repeated offences	First-degree murder; attempted murder; sexual assault; child sex offences	Youth are designated as “serious” or “violent” offenders, while adults are deemed “high-risk” offenders
Officers’ perceptions of offenders under their supervision	Officers view youth as “the worst of the worst,” entrenched in criminality	Officers view adults as “the worst of the worst,” and chronic offenders	No difference in how officers describe individuals under their supervision
How officers negotiate informal rehabilitative support	Voluntary agreements	Voluntary agreements	No difference. Both programs require individuals to demonstrate genuine desire to “want help”

The data analysis was guided by discourse analysis. This approach allows for investigating the types of language participants use when describing their experiences (Johnstone, 2017). The semi-structured interview and focus group formats enabled participants to discuss their experiences openly and offer insight into how they conduct their operations. It also allowed the interviewers to ask follow-up questions and better understand how policies translate into practice.

After data collection and transcription, the analysis began with a thorough review of the interviews. Using the QSR NVivo software, interview responses were thematically coded under several categories. The codes were developed through knowledge from existing literature and the participants’ responses. Several initial codes were established, including: “monitoring strategies,”; “perceived role of officers,”; “relationship building with offenders,”; “informal rehabilitative support,”; “formal rehabilitative support,” and “background/context.” Sub-codes were also created to separate and compare responses between the youth and adult ISPs. The codes were iteratively reviewed and refined during the framing and writing of the analysis.

## The Sites

### Youth Program

According to Canada’s Youth Criminal Justice Act (YCJA), youth charged or convicted in youth justice courts may be placed under a supervision order into intensive support or supervision programs. The YCJA specifies that young offenders can be placed under intensive supervision anywhere between 2 and 7 years, depending on the severity of the offence committed. Operating under the YCJA, the youth program currently manages young offenders who are considered at high risk of reoffending. The age range of the youth in this program is between 12 and 24. However, one officer explained that the youngest person admitted, to the best of their knowledge, was 14 years old. Although the YCJA’s provisions are intended explicitly for youth between 12 and 17, those who are 17 at the time of offence or sentencing can still be admitted into the program and remain under intensive supervision for upwards of 7 years. In this program, the youth are designated as either “serious” and/or “violent” offenders. According to the ISP officers, the typical youth in this program has been arrested and convicted of severe drug-related crimes, assault, armed robberies, or repeated offences. When this research was conducted, the organization comprised 10 police constables, one crime analyst, and one sergeant who oversaw the entire operation. Paired into partnerships of two, each individually monitors 20 to 25 youth, roughly adding up to 120 to 150 in the program at any given time. To be released from the program, one must desist from all criminal behavior and technical violations of conditions for a given time frame, which is usually decided by the sergeant or a court ruling upon (or before, in the case of the court ruling) entry to the program. If a youth exceeds 24 and still engages in criminal activity, they are transferred to the adult ISP.

### Adult Program

The offenders in the adult program are designated as high-risk offenders—a slightly different designation than the “serious” and/or “violent” offenders in the youth program. The adult program only monitors high-risk individuals on probation, on conditional release from jail, or those that meet the parameters for the courts to impose a peace bond under section 810 of the *Criminal Code of Canada*.^6^ The offenders admitted to the adult program are generally convicted of more serious crimes such as first-degree murder, attempted murder, or sexual assault, including child sex offences. To be released from the program, the high-risk offender must desist from all criminal activity for 1 year and complete all of the supervision orders mandated by the court. The adult unit includes five police constables, a crime analyst, and a sergeant. During the interviews, however, the current sergeant was retiring, and a new sergeant was transferring over to the program; we interviewed both. The adult program manages around 20 to 25 adult offenders at any given time, including those currently in remand. Each police officer oversees three to four offenders but is not exclusively assigned to any of them. Moreover, due to the comparatively small police-offender ratio, the officers are familiar with the offenders and their family members, friends, and intimate relationships.

## Be Good, or We’ll “Put You in Jail”: Compulsory Persuasion to Rehabilitate

The primary directive in the youth and adult ISPs is to limit the instances of severe and/or violent crimes committed by high-risk offenders under their supervision. These programs use enforcement strategies to achieve this goal while providing individualized support. Notably, nearly all the participants emphasized that public protection and incapacitation are paramount. Alden, the acting sergeant for the youth program, elaborates on this point, arguing that the youth under their supervision are not the typical offenders under probation or parole:

It’s not like we’re just [monitoring] John’s kid, Dave’s kid over here. We’re talking about criminals. They are youth, but they are criminals. They are entrenched at the point that we get them. They’re not someone that you just think, “Hey, this person will be okay. Just needs a little smack on the wrist.” It’s not like that. This person’s probably already been in jail for a while, this person wants to commit a crime, this person is committing a crime. . . And so [when] we say serious and habitual offenders, we mean youth that have serious criminal convictions, not just charges, not just suspects but are convicted of serious crimes, multiple serious crimes and have a pattern of offending. So, we get the worst of the worst. (Alden, youth program acting sergeant)

The acting sergeant’s sentiment here reflects existing literature that highlights the process of “adulteration” for youth who are convicted of serious crimes (see Goldson & Muncie, 2006; see also R. Miller & Applegate, 2015; Myers, 2003; Phoenix & Kelly, 2013; Singh, 2023). Although their age still reflects that of a minor, their histories, according to the sergeant, entrench them into a perpetual cycle of criminality where a youth “wants to commit a crime.” The youth who enter this program are considered the “worst of the worst”—a type of rhetoric used to rationalize treating them as adults and justify their placement in the program. Adrienne, the youth program’s crime analyst, echoes the acting sergeant’s points:

Like it’s not somebody that stole a chocolate bar; they’ve been through all the different systems. Every probation has had a try at them. Child welfare tried assisting them. Everybody’s tried to put them on the right path, and they still decide that that’s not the right path. So, then we get them. (Adrienne, youth program crime analyst)

Reiterating how entrenched the youth are in criminality, the crime analyst places the responsibility of being on “the right path” onto the youth. The language of the crime analyst indicates that the failure of not having been rehabilitated falls on the youth rather than the various probation and child welfare programs. This type of discourse is common among youth justice workers and highlights a more neo-liberal approach to rehabilitation (Adorjan & Ricciardelli, 2018; Muncie, 2005; Phoenix & Kelly, 2013). The youth here are told that they decide to go on “the right path” and that they are the primary agents to seek change in their lives, despite this being the purpose of some programs like probation or child welfare.

The notion of being entrenched in criminality is a common theme across the youth and adult ISPs. Cornell, a constable from the adult program, uses the same phrasing as the youth program’s acting sergeant when describing the typical high-risk offender they monitor. They state:

We do the worst of the worst people. Like every one of our guys has either killed somebody, tried to kill them, or have done repeated sexual assaults on children. That’s our world. (Cornell, adult program constable)

This constable also characterizes the high-risk offenders under their supervision as “the worst of the worst.” Although both ISPs use identical phrasing, there is a substantive difference between the types of crimes committed by the youth and adults. Compared to the youth program, where the typical high-risk offender is convicted of repeated drug-related offences, assaults, or robberies, the adult program is responsible for individuals convicted of homicide, attempted homicide, or sexual assaults on children. The high-risk label, thus, in this context, does not describe one type of offender but is instead a broad term that captures a wide array of offending. Despite the substantive differences, the underlying commonality is that both programs understand that high-risk offenders are entrenched in criminality. Yet, none of the participants across either program argue that the high-risk label precludes individuals from receiving rehabilitative support. Officers discuss that they still try to help, but their approaches almost entirely depend on the high-risk offender.

Upon being placed in the program, the assigned supervising officer(s) meets with the high-risk offenders to determine an initial monitoring strategy, including whether and how they can provide rehabilitative support. Monitoring strategies refer to the approach officers undertake when enforcing the formal conditions imposed by a court.^7^ In both programs, the monitoring strategy is usually decided shortly after the first interactions between the high-risk offender and the supervising ISP officer. Alden, the acting sergeant of the youth program, explains:

[W]hen we go, and we introduce ourselves to the youth, we’ll just say “we are constable so and so, so and so for [youth program]. You now are part of us, we’re going to be monitoring your conditions.” And we kind of give them a spiel, and it’s really laid back. And we just kind of joke around a bit and just kind of tell them how we can help them if possible. . . We’re pretty upfront and honest. We’re like, “If you go back out and do a crime, we’re going to put you in jail.” At this point, they know, we make it very clear we are the police still, so we’re still going to be that part of your life that’s going to put you in jail. (Alden, youth program acting sergeant)

This sergeant’s statement reemphasizes the primary purpose of the ISP: to ensure that high-risk offenders desist from criminality. Despite the informal tone, the initial meeting is coercive to induce offender compliance. The officers clarify that the youth are responsible for choosing their path. For youth who “go back out and do a crime,” the officers will act on the threat of incapacitating them. Officers offer to “help them if possible,” but there is a clear emphasis on using enforcement strategies. However, the coercive approach toward young offender responsibilization does not radically transform them into law-abiding citizens (Phoenix & Kelly, 2013), especially in the case of high-risk offenders.

Cornell, an adult program officer, facilitates the initial meeting similarly to the youth program’s acting sergeant. Emphasizing deterrence and incapacitation, they state:

So, at the end of the day, our job is really to hold these people accountable if they’re not trying to get help. And I think in real simplistic words, what we usually tell them is, “my job is to stop you from reoffending. I don’t care how we do it; either I throw you in jail, or you stop. I’ll help you either way.” And that’s kind of the parameters we set down for ‘em, as simple as it is. (Cornell, adult program constable)

This officer dispassionately explains that their primary duty is to stop high-risk offenders from committing crimes. Similar to the stance of the acting sergeant, this officer’s strategy is enforcement-oriented, where they explain to the high-risk offenders that they will admonish them if they are “not trying to get help.” Although opportunities for rehabilitative support are mentioned in passing, both ISPs emphasize the importance of their role in public protection and crime prevention; to them, the high-risk offender either “stops” or gets “thrown in jail.”

Another youth program officer explains how they approach their initial meeting. Notwithstanding this officer’s coercion, they emphasize that they intend to provide support—but only if they perceive the high-risk offender actively desists from crime under their supervision.

I tell them, it’s your life, it’s your choice. I’m going to handle you how you want to be handled. If you want to be a criminal and you want to commit crime, then you’re going to see me a lot, and I’m going to deal with you this way. If you want help, and you’re going to follow the law, you’ll deal with me less, and I’m going to help you with what you need to go that way. And ultimately, at the end of the day, it’s your choice. I’m not here to make a decision for you. You tell me which way you want to go. Your actions are going to tell me which way you want to go. (Horace, youth program constable)

This youth program officer also highlights how they place the responsibility on the high-risk offender to seek out support independently. They are willing to help but emphasize that “it’s your life, it’s your choice.” According to the officers, the extent of support provided is entirely dependent on the type of relationship established with the high-risk offender. The officers argue that the high-risk offenders’ words and actions are integral to providing support.

Although the tone of the initial meetings indicates that rehabilitation is not a priority, Jerry, the outgoing sergeant from the adult ISP, explains that officers do express the desire to support the high-risk offenders. They explain:

One of the things we like to try to do, because there are so many offenders that could meet the parameters here, and we have, again, finite resources, is we really look at the—if the offender has any inclination to rehabilitate, we use that as a huge “let’s move them forward” type motivation because lotta these guys, we know they’re not going to change, so all we’re really doing is taking some of the—helping the streets so that they’re not being totally inundated with these guys, but ideally, what we want to be able to do is rehabilitate these guys. (Jerry, outgoing adult program sergeant)

For the adult ISP, the high-risk offender’s inclination to rehabilitate, according to the sergeant, is a significant consideration in whether they accept them into the program. The sergeant attributes this practice to resource constraints, an oft-cited factor in the disjuncture between the ideal goals of rehabilitation and the reality of implementation (Adorjan & Ricciardelli, 2018; Halushka, 2017; Lynch, 2000). With limited resources and funding available to support high-risk offenders, the adult ISP must be selective about who is brought into the program—namely, those inclined to rehabilitate. For youth and adults alike, the onus is entirely placed into their hands to demonstrate a meaningful desire to rehabilitate. Consequently, there is an implicit hierarchy between the types of high-risk offenders in the two programs: those who “choose” to continue to commit crime and those who present a vested interest in their rehabilitation.

## “Voluntary Agreements”: Negotiating and Providing Informal Rehabilitative Support

According to the participants, rehabilitation is possible through two means: enforcement of court-mandated conditions and informal rehabilitative support without enforceable conditions. The primary focus of this analysis is on the latter. As discussed in the previous section, the ISP officers place the onus to rehabilitate in the hands of the high-risk offender. Depending on the level of perceived authenticity, the officers may decide to take a more active role in supporting them. The crime analyst from the adult ISP refers to *voluntary agreement* as the mechanism through which informal rehabilitative support is facilitated. Lucinda, the crime analyst for the adult program, elaborates:

[B]ecause they are chronic, that’s their way of life. Lots of these guys are addicted to drugs and alcohol, socially inept, they just—that’s the only thing they know. So, we do try to encourage them to take part in programs. Sometimes it’s court imposed, sometimes it’s, you hope for voluntary agreement, but that doesn’t always happen. They’re always very willing when you speak to them in remand to take part in programs, and then upon release, I always extend the offer and encourage them to contact me when they are released. . .I have had some contact me and communicate for a little while, but then typically, you see their behaviour changing. From the time they get released, I feel some of them genuinely do want to give it an honest try, but then once they are released, they reacquaint with old friends and the old lifestyle, and it’s very quick for them to take that slide. (Lucinda, adult program crime analyst)

Through discussing their personal experience, the crime analyst explains that voluntary agreements are the only other mechanism through which they can provide support for high-risk offenders. However, officers in both programs acknowledge the difficulty of following *voluntary agreements*. Since voluntary agreements are tacitly reached between the high-risk offender and the ISP, these agreements are effectively unenforceable. Instead, the ISP officers’ coercive threats of incarceration operate as the enforcement mechanism in this context. With coercion as the underlying means to achieve compliance, the high-risk offender is responsible not only for initially seeking out support but also for maintaining efforts. Otherwise, they are or being subject to incapacitation by the supervising officer. The offender responsibilization approach is undertaken even though the crime analyst admits that the “chronic” circumstances of high-risk offenders lead to a quick “slide” back to the “old lifestyle” after being released from remand.

To the officers, compliance, regardless of how it is achieved, is essential to successful rehabilitation through voluntary agreements. Many of the officers in both ISPs stress how, without it, neither formal nor informal rehabilitation measures can be actualized. Renee, an officer from the adult program, explains:

You start with cooperation and try to get their life stable. You know, if we need to go that that bad cop route, you might need to go there. But you don’t start with that cause you can’t go from bad cop to good cop. Start out with rapport. Keep it positive. (Renee, adult program constable)

This officer recognizes the importance of their professional demeanour for a successful voluntary agreement. Their disposition reflects studies that highlight how collaborative relationships with high-risk offenders can substantially reduce recidivism rates (Chamberlain et al., 2018; Vanstone, 2013; Weaver & Barry, 2014). This officer also understands that they must maintain a proper rapport with the high-risk offender from the beginning—they must start positive, as they will not be able to switch between “bad cop to good cop.”

Other officers, however, are quick to point out that cooperation is not obtainable with high-risk offenders that they perceive as inauthentic. They believe that if the high-risk offender does not demonstrate a willingness to rehabilitate through their actions, the officer’s rehabilitative efforts will go wasted. The youth program acting sergeant explains:

I can talk and talk and talk, but you don’t want to listen, then I’m not doing anything. So you need that cooperation, you need that want from the youth. If the youth doesn’t want to be rehabilitated, if the youth doesn’t want to make good decisions, I can’t force them. Because normally if the youth wants it. . . and I know of the coppers here, we’re not hard done by, you can’t be hard done by in this job. So you can call me a fucking pig, a fucking loser, you’re going to rip my throat off one day. And the next day, if you come to me and say, “hey, could I get a little help doing this.” I’ll be there. I don’t take things personally. Nobody on my team takes things personally. And at any point somebody comes to us and says, “Yeah, I want some help,” guaranteed they’ll be like, “Yeah, absolutely, how can we help you?” But at the same time, if it’s like you’ve burnt the bridges many and many times, and this is just a delay tactic, then we get that too. We’re not idiots. (Alden, youth program acting sergeant)

The youth program sergeant reiterates the importance of compliance on behalf of the high-risk offender. With it, the officer is inclined to provide rehabilitative support. This sentiment is amplified if the officer believes the high-risk offender is deceitful in their intentions or solely agreeing to rehabilitate as a “delay tactic.” Suppose the officer perceives the high-risk offender as genuine. In that case, they will be less likely to take negative interactions personally and more likely to offer support upon the high-risk offender’s request. For this reason, the youth program’s crime analyst explains why they decide to wait until the high-risk offender comes to them before they discuss any voluntary agreement. They elaborate:

They’ll come to [us] when they need help, and we’ll help. We’ll arrest them, and we’ll take them to jail when we need to. But, usually, when they’re out, they won’t tell us anything. When they go to jail, [our officers] are the only ones that go to visit them. So, we’re almost their mom or dad. And then when they’re out it, it’s like, “I don’t want anything from you guys, I’m fine, everything’s good.” And then they’re arrested the next day. Like, really? When they’re in trouble, it’s like, “Ah, something happened.” It’s exactly a mom-dad relationship. (Adrienne, youth program crime analyst)

The perceived authenticity of a youth is difficult to interpret while incarcerated. When officers visit them in jail, they appear genuinely interested in getting help, but upon release, they decline any support from the ISP officers. Notably, this crime analyst refers to the relationship between the youth and the officers as parental or familial. They understand that the youth occupy two broad spaces—one of the entrenched criminal and the other of a minor, where they require a form of pseudo-parental guidance. With this in mind, officers from both programs acknowledge that the lack of follow-through on voluntary agreements is not always disingenuous.

[Sometimes] they want help or at least give the perception that they want to try to change things, but they’re kinda stuck in that middle. Sometimes its gang related. . . like they don’t want that lifestyle, but they’re so entrenched in it that they’re having trouble getting out, and you then tailor the program, at least in my opinion, you tailor it to that combination of enforcement and investigating and then trying to assist them with programs or assist them even with support. A lot of time, they don’t want a strict program or support structure they just want they just want somebody outside of their world who can kind of guide them or give them some advice. . . (Horace, youth program constable)

This officer discloses how they use discretion to “tailor” their monitoring strategies. Even in cases where they believe the high-risk offender is entrenched in criminality, they can employ a balanced approach by providing rehabilitative support wherever possible. In this case, the officer believes that sometimes the high-risk offenders do not desire a formal or strict support mechanism; instead, they seek guidance or advice from an outsider. Although advice and guidance may appear negligible, this type of mentorship can effectively change the high-risk offender’s circumstances (DuBois, 2022; Weinrath et al., 2016).

Concerning informal rehabilitative support, officers from youth and adult programs explain how they use their discretion to help the high-risk offenders under their supervision. In correctional contexts, this type of discretion is understood as “soft power,” which Crewe (2011, p. 456) defines as the “sphere of power that makes unnecessary or precedes the use of direct command or coercion.” By establishing and maintaining a good rapport between the officer and the high-risk offender, the high-risk offender benefits significantly—especially concerning the types of informal rehabilitative support they could receive. Octavia, an officer from the youth program, explains how they provide informal guidance and other types of support. They state:

Simple things. Going to jail to visit them. Some of them we’ll pick up from jail, give them a ride where they need to go. Buy them a meal–they haven’t eaten since jail food. Calling and checking in, “Hey, what’s going on?” Offering different things like, “Hey, are you looking for work?” “Do you need help looking for work?” Offering anything. We can get deals on gym passes. Get them to do other things than hanging [sic] with their buddies so they can work on their health. So, offering those cheaper gym passes. I always extend that olive branch. “Hey, if you want to go for a coffee, call me; we can go for a coffee.” Some will take me up on that. (Octavia, youth program constable)

The informal support discussed by this officer appears to have two purposes: the first is to build a genuine, healthy relationship with the high-risk offender, and the second is to learn about the personal circumstances that influence their criminality. Starting with their release from jail, the officer realizes how small acts like giving a ride or buying a meal can help build a trusting relationship. Once the relationship is established with the high-risk offender, the officer gauges the specific types of support that may help prevent them from returning to old habits. In this instance, the officer believes that helping the high-risk offender find a job or get a cheap gym pass may stop them from interacting with their “buddies,” who might influence their criminality.

More importantly, informal rehabilitative support is not limited to guidance or advice for the high-risk offender. Once a relationship is established between the two parties, the impacts of informal support are unlike anything that could exist through formal measures. Arlo, one adult program officer, gives an example:

You know, [name of the high-risk offender] wanted to have another relationship with an adult female who appeared very stable. It’s like how do you tell someone, “You can never have any relationships,” right? So, we sort of looked into this lady and her history, and she had no record. We talked to her about what his history was, and sort of “just so you know, and you can phone us, please phone the police if there’s any problems. . ..” (Arlo, adult program constable)

This ISP officer explains how, after their due diligence, they allowed one of the high-risk offenders under their supervision to develop a new intimate relationship with an adult female. Since an effective way of deterring crime is by changing the personal circumstances of the high-risk offender, this new, healthy relationship could have a lasting positive impact on their recidivism. Despite the tone set in the initial meetings, compassion on behalf of officers is not uncommon within these two ISPs. Another constable from the youth program discusses how they help the high-risk offender and their family. They explain:

It’s all about finding out what makes them tick, right? Knowing who they are, where they come from and then trying to figure out what’s that one thing that gives you a foot in the door. Once you get your foot in the door, then. . . and that could be. . . You know. . . they love their mom, and their mom is their number one thing in the world. Well, okay, I’ll help your mom get a job, or I’ll help your mom do this. “Oh man, I really appreciate it.” And then now you’re in, you know what I mean? (Horace, youth program constable)

This officer explains how they seek to find what makes the high-risk offender “tick.” Through the relationship developed with the high-risk offender, they learned that the mother is a significant figure in their life. The officer then attempts to build the trust further by assisting the mother in finding an occupation. This informal strategy is not at all a part of the explicit duties of this ISP officer, yet by helping the mother of the high-risk offender, they are effectively carrying out their end of the voluntary agreement. This compassionate approach not only gets the officer’s “foot in the door” but can also garner the cooperation or complicity of the high-risk offender.

## Discussion and Conclusion

Examining how informal rehabilitative supports are negotiated and provided by officers in the era of late-modern rehabilitation presents new insights into the role of discretionary decision-making in ISPs.^8^ The reality of our current criminal justice system is that courts do not always impose rehabilitative conditions for those placed in ISPs and that programs face resource constraints that restrict their ability to adequately provide rehabilitative support (Adorjan & Ricciardelli, 2018; Halushka, 2017; Lynch, 2000). Consequently, late-modern rehabilitation focuses on addressing targeted, individual risks to resolve the broader goals of public protection and risk management, while personal rehabilitation is placed by the wayside (Garland, 2001; Halushka, 2017; Hannah-Moffat, 2005; Robinson, 2008). In absence of enforceable conditions, and without officer discretion, there are very limited avenues for which high-risk offenders can receive treatments necessary to desist from crime. As we presented in this study, the discretion employed by officers can uniquely address the personal, familial, intimate, and criminogenic needs of high-risk offenders without the need for enforceable rehabilitative conditions. Translating “soft power” (Crewe, 2011) into the context of ISPs illustrates how officers can greatly enhance both the youth and adult high-risk offenders’ lives and bolster the late-modern era’s emphasis on risk reduction. Yet, the caveat is that only individuals perceived to be inclined to rehabilitate or maintain a positive relationship with their supervising officer will receive such informal support. Reflecting compulsory persuasion, the only choices given to youth and adult high-risk offenders are based on coercion: either desist from criminality or return to jail. Although they may not receive enforceable rehabilitative conditions, the very threat of incarceration is the enforcement mechanism that officers use to motivate a high-risk offender’s rehabilitation. This process hierarchicalizes youth and adult high-risk offenders into ordinate and subordinate groups: those who choose to rehabilitate themselves and those who choose not to rehabilitate themselves. The former group will receive unique, informal rehabilitative support that can drastically change their lives and their families’ lives; the latter group will remain under the intensive supervision of the officers inside and outside of prison until they decide to rehabilitate.

Although existing literature highlights how officers’ interpretations of their responsibilities lead to varying degrees of role ambiguity (Haqanee et al., 2015; Kemshall & Maguire, 2001; Werth, 2018), ISP officers in both programs indicate that their role is primarily that of enforcement and that they will not hesitate to incapacitate youth or adult high-risk offenders who do not desist from criminality. The remarks from these participants support findings from empirical research on the regulation of high-risk offenders (McAlinden, 2010; Ugwudike, 2012; Weaver & Barry, 2014). With high-risk offenders being perceived as entrenched in criminality, officers are less inclined to want to support them in rehabilitation (Ugwudike, 2012). Not surprisingly, there were no differences in how the youth and adult ISPs discussed the high-risk offenders under their supervision. Although research demonstrates that the youth criminal justice system is lenient toward young offenders, young high-risk offenders are not afforded the same considerations (Bolin & Applegate, 2016; Weinrath et al., 2016). The type of language used by participants supports the notion that entrenchment in criminality or committing “adult crime” precludes young offenders from being perceived as youth in the eyes of enforcement (Myers, 2003; R. Miller & Applegate, 2015). As the acting sergeant, Alden said: “They are youth, but they are criminals.” The emphasis on “but” here indicates that one cannot simultaneously be a youth and a criminal. Instead, the label “criminal” overtakes a youth’s identity and dictates how they will be treated by law enforcement.

This study also exemplified what informal rehabilitative support looks like in ISPs. Our research reinforces other studies on the prevalence of mentorship, especially for young, high-risk offenders (DuBois, 2022; Weinrath et al., 2016). Participants discuss how they provide informal guidance and advice to high-risk offenders. Horace, the youth program officer, emphasized how, in some instances, the youth do not seek a formal support structure but rather informal guidance from “somebody outside of their world.” Aside from mentorship, this study documented other supports such as help finding work for the high-risk offender and their families, help attaining recreational gym passes, and help developing a new intimate relationship. Studies find that creating new relationships after being convicted of a crime is difficult (Martinez & Abrams, 2013). The stigma of crime often prevents new meaningful connections from being developed. In the case where the adult ISP officers allowed one of their high-risk offenders to build a new intimate relationship with an adult female, such considerations can have significant impacts on the high-risk offender’s life; healthy, supportive relationships are shown to reduce the likelihood of reoffending (Martinez & Abrams, 2013). In the case of helping the young high-risk offender or their family member to find a job, officers effectively address one of the possible criminogenic factors that can lead to their criminality (Schaeffer et al., 2014). Small actions like this can have a drastic impact on the life of a young offender. Similarly, participation in recreational sports or physical activities can help both youth and adults desist from crime (Van Hout & Phelan, 2014). The officer administering the high-risk offender a gym pass intended to get them away from acquaintances who might influence their criminality. All these informal supports are rooted in the compassion of supervising officers. However, compassion is only afforded to certain high-risk offenders. Although many could benefit from such informal supports to break the perpetual cycle of criminality, only individuals perceived as genuinely inclined to rehabilitate will receive them.

## Limitations and Future Research Directions

Despite this paper’s essential contributions, this study contains several limitations. First, the sample size of our study is small, and the views of the officers from these two ISPs in one Canadian city cannot be interpreted as representative of ISP officers’ views across Canada. Considering the participation among the officers was voluntary, this could introduce bias regarding the responses provided to the researchers. Significantly, our study warrants further investigation on the intensive supervision orders of the Youth Criminal Justice Act, as research on Canadian young offenders under intensive supervision—and in juvenile detention—is largely absent from academic discourse. Considering the stark neglect around any mention of race by the officers in this study, future research should examine whether and how racialized and Indigenous youth under intensive supervision receive informal rehabilitative support in the absence of formalized conditions imposed by a court.

## Notes

1. While recognizing the stigmatizing connotations of “offender” (Willis, 2018), we employ this term throughout our paper. The term “offender” is prominent in the literature on intensive supervision, and our work demonstrates the power of this label over the lives of those under intensive supervision.

2. What qualifies as high-risk varies between geography and jurisdictions, which means that individuals who are deemed high-risk in one region may not meet the threshold of high-risk in another (see Ballucci, 2023). In some cases, the individuals are not charged or convicted of an offence, but are still given this designation and placed under the supervision of ISPs (see Lecoq et al., 2021).

3. Every jurisdiction can have different names for intensive supervision programs. Some examples include prolific/priority offender programs, high-risk offender programs, serious/habitual offender programs. Although the names of the programs differ, they all operate as a form of intensive supervision.

4. Intensive supervision programs vary with respect to the types of officers they employ. Some ISPs operate with correctional officers, probation workers, or parole officers (see Adorjan & Ricciardelli, 2018; Haqanee et al., 2015; McCahill & Finn, 2013). However, the youth and adult ISPs in this study employ variously ranked police constables and detectives, which is also common (see Lecoq et al., 2021; Xing & Ballucci, 2023).

5. Although the adult program monitors those aged 18 and older, at the time of the interviews, officers explained that the youngest offender in the adult program was 25.

6. Section 810 of the Criminal Code of Canada establishes sureties in order to keep the peace. If an individual “fears on reasonable grounds” that someone may harm them, their family, or damage their property, they can file an application for the potential perpetrator to enter a court-imposed recognizance (see Lecoq et al., 2021).

7. Enforceable conditions discussed by the youth and adult ISP officers include periodic home visits, periodic meetings in police buildings or public spaces, electronic monitoring, phone calls or texts, curfew restrictions, prohibition of alcohol or drug consumption, mandating alcohol or drug treatment programs, and/or other measures dictated by a court. Through monitoring strategies, officers have the discretionary power to choose if and how they follow-up with the high-risk offender if any of these conditions are breached.

8. See Table 1 for a comparison between the youth and adult ISPs approaches.
